# Adherence and Acceptability of an Oral Antibiotic Used for the Prevention of Pediatric Urinary Tract Infection in Japan

**DOI:** 10.3390/pharmaceutics13030345

**Published:** 2021-03-06

**Authors:** Jumpei Saito, Sayaka Miyamoto, Mayumi Yamada, Akimasa Yamatani, Fabrice Ruiz, Thibault Vallet

**Affiliations:** 1 National Center for Child Health and Development, 2-10-1 Okura, Setagaya-ku, Tokyo 157-8535, Japan; saito-jn@ncchd.go.jp (J.S.); miyamoto-sa@ncchd.go.jp (S.M.); yamada-ma@ncchd.go.jp (M.Y.); yamatani-a@ncchd.go.jp (A.Y.); 2 ClinSearch, 110 Avenue Pierre Brossolette, 92240 Malakoff, France; thibault.vallet@clinsearch.net

**Keywords:** medicine, acceptability, adherence, granules, pediatric, children, formulation, antibiotic, urinary tract infection, ClinSearch acceptability score test (CAST)

## Abstract

Urinary tract infection (UTI) is a common health care-associated adverse event and the leading nosocomial complication following pediatric urological surgery. While continuous antimicrobial prophylaxis effectively reduces the risk of UTI following such a surgery, non-adherence is common and represents a distinct clinical entity that is associated with renal scarring. Acceptability is likely to have a significant impact on patient adherence. Herein we used a validated data-driven approach—the ClinSearch acceptability score test (CAST)—to investigate the acceptability of cefaclor, an oral antibiotic widely used for the prevention of pediatric UTI in Japan. Standardized observer reports were collected for 58 intakes of cefaclor 10% fine granules in patients aged from 0 to 17 years. The medicine was classified as positively accepted on the acceptability reference framework. According to the percentage of the prescribed dose taken reported at the end of the treatment, patients exhibited good adherence to this well-accepted medicine. Nonetheless, requirements for greater dosing frequency or poor acceptability in certain patients could affect adherence. Acceptability should be established to ensure patient adherence to medicines used for long-term prophylaxis and consequently guarantee the safety and efficacy of the treatment.

## 1. Introduction

Continuous antimicrobial prophylaxis has been a longstanding management strategy for pediatric patients with vesicoureteral reflux (VUR) and urinary tract infection (UTI)—a common health care-associated adverse event and the leading nosocomial complication following pediatric urological surgery [1,2,3,4]. In a randomized interventional trial of pediatric patients with VUR, prophylaxis was associated with a two-fold reduction in risk for recurrent UTIs [5]. Although there is no clear consensus on duration of antimicrobial prophylaxis for VUR [4,6], periods of about one year of continuous prophylaxis have been reported [7,8,9]. Shorter periods of several days continuous antimicrobial prophylaxis treatment are currently recommended after postoperative (open and laparoscopic) pediatric urological surgery in the updated Japanese urological guidelines [10].

Due to potential long periods of treatment, ensuring an adequate adherence is especially challenging for pediatric patients where various factors such as medication taste, dosing frequency or daily life and environment may affect this crucial parameter [11,12]. Thus, the European Medicine Agency has highlighted in a guideline on pharmaceutical development of medicines for pediatric use the importance of patient acceptability, defined as the overall ability and willingness of the patient to use and its care giver to administer the medicine as intended. Patient acceptability is likely to have a significant impact on patient adherence [13].

Adherence to prophylaxis management is reported to be distinctly related to clinical outcomes [14] and non-adherence represents a clinical entity that is associated with renal scarring. As such, best adherence must be maintained for the prophylaxis management for pediatric patients with UTIs. Wide ranges in adherence rates specifically for prophylaxis in pediatric patients with UTIs have been reported, varying from 40 to 90% depending on measurement methods and patient populations [15,16].

While prophylaxis management using low dose cefaclor can effectively reduce the risk of recurrent UTIs [17] and is commonly used, according to our knowledge there is no data regarding its acceptability in Japan. Herein, we investigated patient adherence and acceptability to this common oral antibiotic.

## 2. Materials and Methods

### 2.1. Objective, Study Design and Setting

This monocentric, prospective, longitudinal, and strictly observational study aiming to investigate patient adherence and medicine acceptability was carried out in the National Center for Child Health and Development (NCCHD) of Tokyo (Japan) from June 2019 to October 2020. This study approved by the Ethics Committee of NCCHD (2019-081) was conducted on a voluntary basis in patients less than 18 years old treated with any oral/buccal medicine. To explore acceptability, we used a validated data-driven approach simultaneously considering the many aspects of this multi-faceted concept [18,19,20,21]. This article focuses on findings on cefaclor fine granules.

### 2.2. Data Collection

A standardized web-questionnaire was used by a trained member of the site study team observing the medicine intakes in patients included in the study. In total, nine observable behaviors were recorded for each intake. These included: (1) result of the intake (the required dose was fully, partly or not taken at all); (2) patient reaction during the administration using a 3-point facial hedonic scale (positive, neutral or negative); (3) preparation time (from opening any packaging to having a required dose of medication ready to use, including all handling and modifications), and time to administer the required dose of medication (from a required dose of medication ready to use to the end of the intake). The sum of preparation and administration times was classified as short (1 min and less), medium (from 1 min to 2 min and 30 s), or long (more than 2 min and 30 s). In addition, any of the following methods used to ease/achieve administration were reported resulting in 6 binary variables with two possible values-used or not: (4) dividing the intake of a dose which cannot be taken as a whole; (5) altering the intended use (modifying the dosage form such as tablet crushed or using another route/mode of administration such as oro-dispersible tablet swallowed); (6) using food/drink either mixed with the drug or taken just before or after administration to mask the taste or ease swallowing (using water for granules intake was considered as an intended use and not as a method to help medicine intake); (7) using a device not provided with the medication; (8) using a reward; (9) using restraints (the patient had to be made to take it). Each evaluation of one medicinal product, taken by one patient, corresponded to a distinct combination of observed measures (e.g., neutral reaction) for all nine observational variables listed above (e.g., patient reaction) which incorporate the many aspects of acceptability. Observed measures were collected for the first medicine intake occurring after the patient’s inclusion into the study (timepoint 1), 24 h after (timepoint 2), and for the last medicine intake of the treatment (timepoint 3).

The questionnaire was also completed at timepoint 1 with information on the treatment (exact name of the medicine, the required dosing frequency and duration of treatment) and the patient (sex, age and previous exposure to the treatment).

At the end of the third login, information on adherence were reported: the percentage of prescribed dose taken (0–20%, 21–40%, 41–60%, 61–80% or 81–100%) and any difficulty with preparation and administration of the medicine influencing patient adherence—binary variables with two possible values (yes or no) for both.

### 2.3. Data Analysis

A description of the demographics, acceptability and adherence data was performed. Categorical variables were described by the size and the percentage of each category. Numerical variables was described by the size, the mean and the standard deviation (SD), as well as the minimum and maximum values.

Comprehensive scores were produced using the acceptability reference framework. Multivariate analysis—mapping and clustering—is employed using data collected since May 2015. It was thus possible to mine a large set of 2,611 evaluations comprised of those from Japan as well as further data collected using the same standardized questionnaire in six other countries with various cultures (France, United Kingdom, Germany, Norway, India and Morocco).

Multiple Correspondence Analysis (MCA) highlighted similarities between all the evaluations and the key relationships between the nine observational variables and more precisely associations between the 21 observed measures, into a low-dimensional space: the 3D acceptability map. The three dimensions of the map visualized in an intelligible form those associations and dissociations of observed measures that contributed the most to explaining variability observed in the data. The observed measures and the evaluations were positioned together on the acceptability map. Interpretation of the map is based on the distance between elements. Observed measures closed on the map were often selected together in the evaluations—combinations of 9 observed measures. Similarly, the evaluations were positioned on the map according to their similarity, from the combination of the ideal observed measures to the worst combinations. Subsequently, the most similar evaluations were gathered into clusters using Hierarchical Clustering on Principal Components of the factorial analysis (HCPC) and k-means consolidation. The clusters were characterized by the observed measures significantly over-represented into their subset of evaluations in comparison with the whole dataset (v-test value greater than 1.96). Two meaningful clusters defining coherent acceptability profiles—“Positively accepted” and “Negatively accepted”—have emerged. The distinct profiles were materialized on the acceptability map by green and red zones, respectively.

The medicine of interest—cefaclor fine granules—was positioned on the map at the barycenter of all the evaluations collected in this study. Confidence ellipses surrounding the barycenter for all dimension pairs (1–2, 1–3 and 2–3) defined an area containing its true position with 90% probability if the experiment were to be repeated. The size of confidence ellipses is influenced by the sample size and the homogeneity of the evaluations. At least 30 patients were required to obtain a reliable acceptability score. The medicine will be classified as accepted if the barycenter, along with the entire confidence ellipsis surrounding it, belongs to the green area of the map.

Acceptability score based on evaluations at timepoint 1 will be compared with those based on evaluations collected at timepoints 2 and 3 in order to study acceptability of cefaclor fine granules over time. Longitudinal data—timepoints 2 and 3—were not included in the dataset that gave rise to the acceptability reference framework. These evaluations were included in the multivariate analysis as supplementary individuals which had no influence on the model outcomes. Afterwards, they were positioned on the acceptability map to allow scoring process implementation. Distinct acceptability scores are significantly different if confidence ellipses do not overlap on the map.

Acceptability evaluation has to be relative, and consequently the acceptability score based on all the evaluations of cefaclor fine granules collected in this study was compared to the average score of powder/granules antibacterial acceptability. This acceptability score was reached using 364 evaluations of 81 distinct pharmaceutical products taken by patients aged 0–15 years from the dataset that gave rise to the acceptability reference framework (Table S1).

The influence of age, sex and treatment exposure on acceptability of the medicine of interest was investigated in secondary analysis. All the evaluations of cefaclor fine granules collected in this study were successively partitioned into subgroups according to these variables and acceptability score was obtained for each category.

The video abstract illustrates the mapping, clustering, and scoring processes.

In addition, statistical tests were used to assess the significance of the differences observed between measures for the nine observational variables composing the different acceptability scores. When there was a minimum expectation of five for 80% of the categories of the contingency table without any null expectation, Pearson’s chi-squared test was used; alternatively, Fisher’s exact test was used.

Data analyses were performed using R version 1.0.136© (RStudio Team (2016). RStudio: Integrated Development for R. RStudio, Inc., Boston, MA, US). The R package FactoMineR was used to perform mapping and clustering processes [22]. The R package missMDA was used to handle missing values in multivariate data analysis [23]. Imputation of observed measures took into account both, similarities between evaluations and relationships between the nine observational variables.

## 3. Results

### 3.1. Patients and Treatment

In the acceptability study in Japan, 58 evaluations of cefaclor 10% fine granules intake were collected (cefaclor 10% fine granules by Sawai Pharmaceutical). This pediatric generic product made by Sawai Pharmaceutical has a light orange color—food yellow No. 5 aluminum lake. Its taste is sweet and slightly bitter—sucrose is used as sweetener—with a slight smell of oranges—no detailed information is available for this flavoring.

Longitudinal data were reported for all the patients, with the exception of one for the second timepoint.

The mean age of the 58 patients treated with cefaclor was 5.8 years (SD = 4.2, range 0–17), 72% were boys, and 90% were treated with this medicine for the first time. The mean treatment duration was 5 days (SD = 3, range: 3–14). Most of the patients (76%) took the medicine three times a day, 22% once daily, and the required dosing frequency was five times a day for one patient. All patients were treated using cefaclor for prophylaxis of postoperative UTIs.

### 3.2. Acceptability

At timepoint 1, positively connoted observed measures were reported in most cases (Table 1). The required dose of medicine was fully taken by all patients with the exception of one 5-year-old girl, and only 9% of patients had a negative reaction. There was no alteration of the intended use and no need to reward the children. Only one 5-year-old girl needed to be coerced to take the medicine, and the required dose was too large to be taken as a whole in just three cases. Nonetheless, we observed that the time of preparation and administration was mainly medium (53%) or long (41%), an extra device not provided with the medicine was used for 55% of evaluations (50% oral syringe, 34% dropper, 13% spoon or 3% tumbler), and for 14% of cases, the granules were mixed into food or drink to ease administration (sucrose syrup, jelly, yogurt or green tea).

Each evaluation corresponds to a combination of an observed measure for each of the nine observational variables. The following combination reflecting a medicine taken without problem using a device not provided with the product was the most used (10% of the 58 evaluations): “Fully taken”, “Positive reaction”, “Medium time”, “No divided dose”, “No food drink”, “No alteration”, “Use device not provided”, “No reward” and “No restraint”. The acceptability reference framework allowed us to deal with the 27 distinct combinations used, which reflected different users’ behaviors.

**Figure 1:**
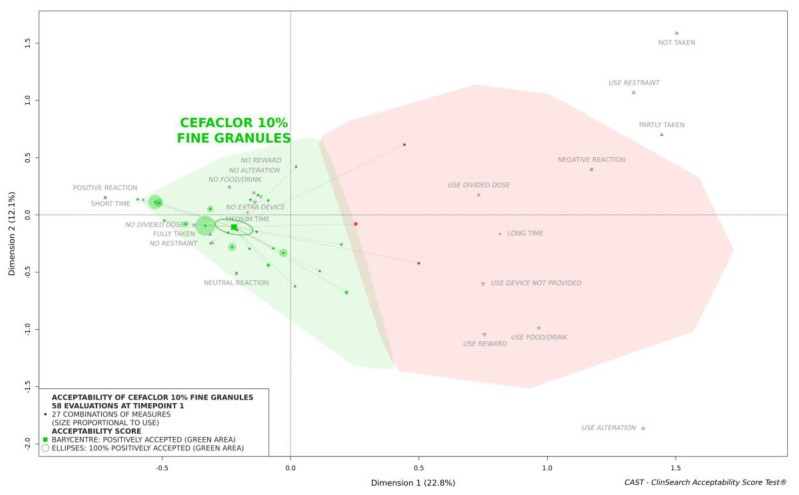
Acceptability at timepoint 1 of cefaclor 10% fine granules.

According to the reference framework, cefaclor 10% fine granules were classified as accepted at timepoint 1: the barycenter of the 58 evaluations, along with the confidence ellipsis surrounding it, was fully located in the green area of the acceptability map (Figure 1).

**Figure 2:**
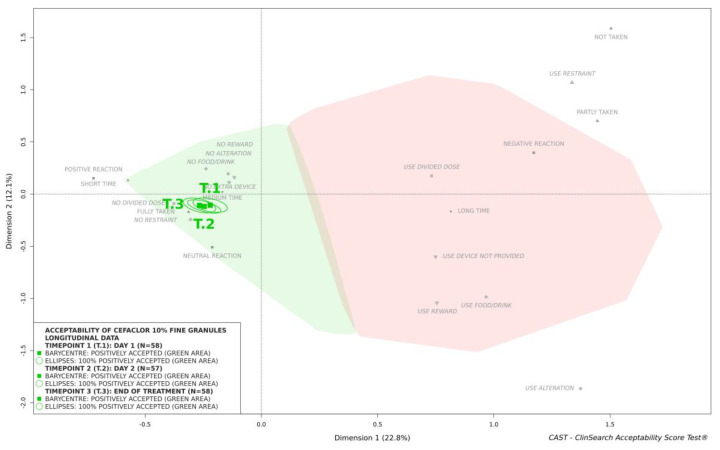
Acceptability over time of cefaclor 10% fine granules.

This medicine was similarly classified as accepted at timepoints 2 and 3 (Figure 2). There was no significant difference over time in terms of the nine constituting observational variables (Table 1) and consequently, the acceptability scores—confidence ellipses overlapped on the acceptability map (Figure 2).

**Figure 3:**
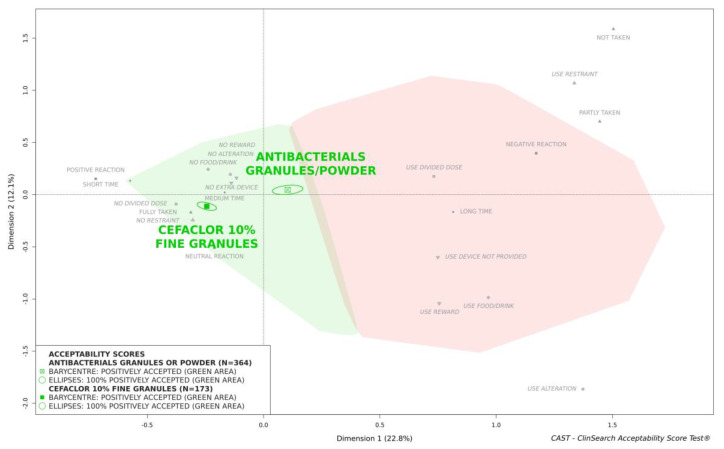
Acceptability of cefaclor 10% fine granules compared with a large scale of antibacterials formulated as powder or granules in children aged 0 to 15 years.

Figure 3 shows that the overall acceptability score of cefaclor fine granules was located significantly further from the negative area materialized in red on the right of the map than the score which reflected the average acceptability of all antibacterials formulated as powder or granules tested in pediatrics. This difference between these acceptability scores reflected significant differences for the nine of the constituting variables.

**Figure 4:**
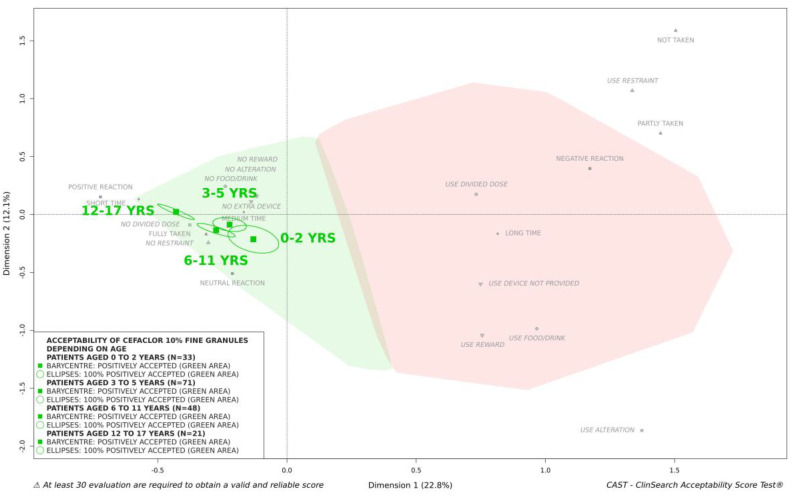
Influence of age on acceptability of cefaclor 10% fine granules.

In secondary analyses, we investigated the influence of the characteristics of the patients on acceptability of cefaclor 10% fine granules. There were less than 30 patients in some categories, sex (16 girls and 42 boys), age (11 patients aged 0–2 years, 24 aged 3–5 years, 16 aged 6–11 years, 7 aged 12–17 years), and treatment exposure (first exposure to treatment for 52 patients, previous exposure for 6), thus data partitioning was performed regardless of timepoints. The medicine under investigation appeared to be positively accepted for each category—the barycenter as well as the confidence ellipses surrounding it were fully located in the green area of the map in each case. Boys and girls were plotted very close on the map, as well as first exposure and previous exposure to treatment. We should note that there were few evaluations (*n* = 18) for the latter category. Regarding patient age, the group of adolescents appeared to be located closest to the ideal position on the map, while the infants and toddlers group was positioned closest to the negative zone in red (Figure 4). Differences between acceptability scores appeared to be mainly driven by the reactions of patients and the use of a device not provided to help with taking the medicine (Table 2). However, cefaclor 10% fine granules were classified as “positively accepted” in all age groups (Figure 4).

### 3.3. Adherence

Among the 56 patients with information on treatment adherence reported at the third timepoint, 96% of patients took 81–100% of the prescribed doses.

Only two patients took only 61–80% of the prescribed doses. This included the 3-year-old boy who was obliged to take the medicine five times a day. Difficulty with medication intake was the reported reason for the imperfect treatment adherence for the other child, a 5-year-old girl—this reason was reported for only two patients. At timepoint 1, this young girl had a negative reaction, the administration took a long time, and the required dose of medication was not taken at all. This evaluation was positioned at the top right of the acceptability map (Figure 1). Nonetheless, for this young girl acceptability did improve over time.

## 4. Discussion

Herein using the acceptability reference framework we demonstrated that cefaclor fine granules are positively accepted in pediatrics in Japan over the course of treatment. The acceptability score of cefaclor fine granules is significantly better than the score which reflected the average acceptability of antibacterials formulated as powder or granules. The latter score was based on 364 evaluations of various products other than the medicine under investigation. These evaluations collected in six countries are a part of the dataset that gave rise to the acceptability reference framework which enables the production of relative acceptability evaluations.

The acceptability reference framework simultaneously considered nine observational variables describing the many aspects of acceptability. Herein, positively connoted observed measures were reported in most cases resulting in positive acceptability scores. However, the use of a device that was not provided with the products (oral syringe, dropper, spoon or tumbler) to help with taking the medicine was reported for more than half of the patients, especially among younger patients. As the use of inappropriate dosing or administration devices may result in undesirable consequences due to dosing errors, this finding underlines the need to provide medicine’s users with an age-appropriate device when appropriate. Although some patients and their caregivers reported a slightly sweet taste, others were obliged to use food or drink other than water (sucrose syrup, jelly, yogurt or green tea) to help with taking the medicine. Co-administration with food or drink is a common practice in pediatrics to overcome palatability or swallowability issues. However, such a strategy to administer drugs to children may have a significant effect on the bioavailability and therapeutic effect of certain drugs. The patient information leaflet of the medicine under investigation stated “treat directly or dissolve with water.” In pediatrics, the recommendation co-administration strategies in the patient information leaflet would be of great interest. In this study, most of the reported patient reactions were neutral or positive suggesting that palatability, which includes taste as well as texture, of these fines granules ranging from 75 to 200 µm, was not a barrier to administration. On another note, while the dosing volume is likely to affect acceptability, this did not seem to be an obstacle for cefaclor fine granules. The required dose of medication was indeed rarely divided to achieve administration. In routine practice, pharmacists from the NCCHD recommended to use 2–5 mL of medium depending on the amount of granules required.

Highlighting good acceptability of cefaclor fine granules, these findings are consistent with referenced studies carried out in Israel and the United States. Using different graded scores reported by phone by the parents of 546 children receiving amoxicillin, cefaclor, cefuroxime axetil or trimethoprim/sulfamethoxazole, Dagan et al. [24] investigated variations in acceptability of those common oral antibiotics. According to the patient acceptance score—”How does your child accept the drug?” (with pleasure; with no problems; with slight resentment; with strong resentment; refused to take the drug)—and the parents’ general satisfaction—”How satisfied are you with the drug in general?” (extremely satisfied; satisfied; partially satisfied; dissatisfied; extremely dissatisfied)—the drugs were classified as follows, from the best to the most poorly accepted: cefaclor, amoxicillin, trimethoprim/sulfamethoxazole and cefuroxime axetil. Based on the reactions of 377 children to medication tastes reported by parents, Pichichero et al. [25] similarly highlighted a higher taste acceptability for cefaclor in comparison with amoxicillin-clavulanate potassium and cefuroxime axetil. Although cefaclor seems to be well-accepted across different cultures, there are some variations in product formulation between countries. In Japan, all pediatric generic products as well as the first marketed cefaclor product (Innovator) are pale yellow fine granules with a smell of oranges. However, cefaclor may be formulated with different flavor and color in other countries. In France, for example, cefaclor fine granules are strawberry flavored. In the United-States, these strawberry flavored cefaclor fine granules could be any color from white to yellowish or red. Further investigations into the influence of different flavors or colors on the acceptability of cefaclor and, more broadly, of antibiotics across different countries, are essential to better facilitate the choice of an appropriate formulation for each specific population.

In this study cefaclor fine granules appeared to be well-accepted in all age groups, even in infants and toddlers. Important acceptability issues have been highlighted in those young patients using the same standardized assessment method in other culture [20]. Further explorations are needed to better understand how medicine acceptability may vary across countries depending on sociocultural aspects.

As all of the recruited pediatric patients in this study were treated using cefaclor for prophylaxis of postoperative UTIs, the treatment period ranged from 3 to 14 days in accord with the updated Japanese guidelines on the prevention of perioperative infections in the urological field [10]. Although this treatment duration is brief compared to the recommended continuous antimicrobial prophylaxis for VUR [7], continuous administration over the adequate period of time is effective for the prevention of pediatric UTI in these cases [26,27]. This work is limited by its exclusive focus on cefaclor. Indeed, for this indication physicians are able to choose from a range different antimicrobials such as amoxicillin/clavulanic acid, second (cefuroxime, cefprozil) and third (cefixime, cefpodoxime, ceftibuten, cefdinir) generation cephalosporins, and trimethoprim-sulfamethoxazole (ST-mixture) [28]. Like Cefaclor, the ST-mixture cited above is commonly used in Japan due to local antimicrobial susceptibilities [29], but its bitter taste and gritty texture seem to result in acceptability issues [30,31]. Future studies on alterative medications will help healthcare professionals account for the acceptability each product in their decision making, and thus prescribe the product best suited for each of their patients.

In this study, patients exhibited good adherence to this medicine that was well accepted over time. These results establish this formulation of cefaclor as appropriate for prophylaxis treatment in pediatrics in Japan. Nevertheless, patient adherence may have been affected by requirements for greater dosing frequency or by poor medicine acceptability in certain individuals. As acceptability of medicines used for long-term prophylaxis seems to be a key factor in pediatric, treatments should be studied and their acceptability confirmed to ensure patient adherence and consequently guarantee the safety and efficacy of any such long-term treatment.

## Figures and Tables

**Table 1:** Observer-reported outcomes over time.

Outcomes	Timepoint 1 (*n* = 58)	Timepoint 2 (*n* = 57)	Timepoint 3 (*n* = 58)	Statistical Test
**Result intake**				
Fully taken	57 (98) ^a^	57 (100)	57 (98)	F ^b^: *p* = 1
Partly taken	0 (0)	0 (0)	0 (0)	
Not taken	1 (2)	0 (0)	1 (2)	
**Patient reaction**				
Positive reaction	32 (57)	31 (55)	35 (60)	F: *p* = 94
Neutral reaction	19 (34)	21 (38)	20 (34)	
Negative reaction	5 (9)	4 (7)	3 (5)	
*missing data*	*2*	*1*		
**Preparation and administration time**				
Short time	3 (6)	8 (16)	7 (14)	χ^2 c^: *p* = 38
Medium time	26 (53)	23 (46)	29 (58)	
Long time	20 (41)	19 (38)	14 (28)	
*missing data*	*9*	*7*	*8*	
**Divided dose**				
No divided dose	55 (95)	55 (96)	57 (98)	F: *p* = 7
Use divided dose	3 (5)	2 (4)	1 (2)	
**Food/drink**				
No food/drink	50 (86)	49 (86)	50 (86)	
Use food/drink	8 (14)	8 (14)	8 (14)	
**Alteration**				
No alteration	58 (100)	57 (100)	58 (100)	
Use alteration	0 (0)	0 (0)	0 (0)	
**Extra device**				
No extra device	26 (45)	26 (46)	25 (43)	χ^2^: *p* = 96
Use device not provided	32 (55)	31 (54)	33 (57)	
**Reward**				
No reward	58 (100)	57 (100)	58 (100)	
Use reward	0 (0)	0 (0)	0 (0)	
**Restraint**				
No restraint	57 (98)	56 (98)	57 (98)	
Use restraint	1 (2)	1 (2)	1 (2)	

^a^ n (%): number and percentages; ^b^ F: Fisher’s Exact Test; ^c^ χ2: Pearson’s Chi-squared Test.

**Table 2:** Observer-reported outcomes depending of age group.

Outcomes	0–2 years (*n* = 33)	3–5 years (*n* = 71)	6–11 years (*n* = 48)	12–17 years (*n* = 21)	Statistical Test
**Result intake**					
Fully taken	33 (100) ^a^	68 (96)	48 (100)	21 (100)	F ^b^: *p* = 45
Partly taken	0 (0)	0 (0)	0 (0)	0 (0)	
Not taken	0 (0)	3 (4)	0 (0)	0 (0)	
**Patient reaction**					
Positive reaction	6 (20)	39 (57)	30 (62)	21 (100)	χ^2 c^: *p* < 001
Neutral reaction	21 (70)	21 (31)	15 (31)	0 (0)	
Negative reaction	3 (10)	8 (12)	3 (6)	0 (0)	
*missing data*	*3*	*3*			
**Preparation and administration time**					
Short time	0 (0)	6 (9)	0 (0)	3 (25)	χ^2^: *p* = 009
Medium time	21 (70)	30 (46)	24 (62)	3 (25)	
Long time	9 (30)	29 (45)	15 (38)	6 (50)	
*missing data*	*3*	*6*	*9*	*9*	
**Divided dose**					
No divided dose	30 (91)	65 (92)	48 (100)	21 (100)	F: *p* = 07
Use divided dose	3 (9)	6 (8)	0 (0)	0 (0)	
**Food/drink**					
No food/drink	24 (73)	65 (92)	42 (88)	18 (86)	χ^2^: *p* = 08
Use food/drink	9 (27)	6 (8)	6 (12)	3 (14)	
**Alteration**					
No alteration	33 (100)	71 (100)	48 (100)	21 (100)	
Use alteration	0 (0)	0 (0)	0 (0)	0 (0)	
**Extra device**					
No extra device	12 (36)	30 (42)	18 (38)	18 (86)	χ^2^: *p* < 001
Use device not provided	21 (64)	41 (58)	30 (62)	3 (14)	
**Reward**					
No reward	33 (100)	71 (100)	48 (100)	21 (100)	
Use reward	0 (0)	0 (0)	0 (0)	0 (0)	
**Restraint**					
No restraint	33 (100)	68 (96)	48 (100)	21 (100)	F: *p* = 45
Use restraint	0 (0)	3 (4)	0 (0)	0 (0)	

^a^ n (%): number and percentages; ^b^ F: Fisher’s Exact Test; ^c^ χ2: Pearson’s Chi-squared Test.

## Data Availability

Data underlying the study cannot be made publicly available due to legal and ethical considerations. European Union (GDPR) and French (Law n˚78–17 of 6 January 1978) laws restrict the public sharing of personally identifiable data. Requests for data will be processed according to the French MR-003 Code of conduct by the data controller, ClinSearch, which allows for the use of data for the purpose of reproducing study results. Requests to access the data for this purpose may be sent to the data protection officer of ClinSearch: dataprivacy@clinsearch.net and researchers outside the European Union will need to sign a transfer agreement.

## Supplementary Materials

The following are available online at https://www.mdpi.com/1999-4923/13/3/345/s1. Table S1. Patient and medicine characteristics of the 364 evaluations of antibacterials formulated as powder or granules in children aged 0 to 15 years within the dataset that gave rise to the acceptability reference framework.
