# Supplementary material for: Adherence and Acceptability of an Oral Antibiotic Used for the Prevention of Pediatric Urinary Tract Infection in Japan
Source: Pharmaceutics. 2021 Mar 6;13(3):345. doi: 10.3390/pharmaceutics13030345 (PMC8000562; doi:10.3390/pharmaceutics13030345)
Supplement: Supplementary file 1 [file pharmaceutics-13-00345-s001.pdf]

pharmaceutics

Supplementary Materials: Adherence and Acceptability of an Oral Antibiotic Used for the Prevention of Pediatric Urinary Tract Infection in Japan

Jumpei Saito, Sayaka Miyamoto, Mayumi Yamada, Akimasa Yamatani, Fabrice Ruiz * and Thibault Vallet

Table S1. Patient and medicine characteristics of the 364 evaluations of antibacterials formulated as powder or granules in children aged 0 to 15 years within the dataset that gave rise to the acceptability reference framework.

Patients and Medicines Characteristics	*n* (%)
**Sex of patients**	
Girl	160 (45)
Boy	194 (55)
missing data	10
**Age group of patients**	
0 to 2 years	162 (45)
3 to 5 years	118 (33)
6 to 11 years	66 (18)
12 to 17 years	11 (4)
missing data	7
**Treatment exposure**	
Previous exposure	159 (44)
First exposure	203 (56)
missing data	2
**Type of care**	
Outpatients	228 (63)
Inpatients	136 (37)
**Country**	
France	197 (54)
Morocco	88 (24)
Norway	42 (12)
England	17 (5)
Germany	12 (3)
Japan	8 (2)
**5th level (chemical substance) of the Anatomical Therapeutic Chemical (ATC) classification system**	
Amoxicillin	87 (24)
Co-amoxiclav	81 (22)
Josamycin	49 (14)
Cefpodoxime	45 (12)
Cefixime	45 (12)
Phenoxymethylpenicillin	27 (7)
Clarithromycin	9 (3)
Cefalexin	8 (2)
Azithromycin	4 (1)
Erythromycin	3 (1)
Clindamycin	3 (1)
Cefaclor	2 (0.5)
Cefadroxil	1 (0.5)

**Dosage form categories**	
Powder	243 (67)
Granule	121 (33)
